# Distribution of virulence genes and phylogenetics of uropathogenic *Escherichia coli* among urinary tract infection patients in Addis Ababa, Ethiopia

**DOI:** 10.1186/s12879-020-4844-z

**Published:** 2020-02-07

**Authors:** Belayneh Regasa Dadi, Tamrat Abebe, Lixin Zhang, Adane Mihret, Workeabeba Abebe, Wondwossen Amogne

**Affiliations:** 1 Department of Medical Microbiology, Arba Minch University, Arba Minch, Ethiopia; 2 Department of Microbiology, Immunology and Parasitology, Addis Ababa University, Addis Ababa, Ethiopia; 3 Department of Epidemiology and Biostatistics, Michigan State University, East Lansing, USA; 4 Department of Pediatrics, Addis Ababa University, Addis Ababa, Ethiopia; 5 Department of Internal Medicine, Addis Ababa University, Addis Ababa, Ethiopia

**Keywords:** Urinary tract infections, Uropathogenic *Escherichia coli*, Virulence genes and phylogroup

## Abstract

**Background:**

Urinary tract infection (UTI) is a common cause of morbidity worldwide. Uropathogenic *Escherichia coli* (UPEC) bacteria are the major cause of urinary tract infections. UPEC strains derive from different phylogenetic groups and possess an arsenal of virulence factors that contribute to their ability to overcome different defense mechanisms and cause disease. The objective of this study was to identify phylogroup and virulence genes of UPEC among urinary tract infection patients.

**Methods:**

A cross sectional study was conducted from January 1, 2017 to October 9, 2017. *E. coli* bacteria were isolated from UTI patients using culture and conventional biochemical tests. Identification of phylogroup and genes that encodes for virulence factors was done using multiplex polymerase chain reaction (PCR). Data was processed and analyzed with SPSS version16.0 and Epi-info version 3.4.1 software.

**Results:**

The most common urologic clinical manifestation combinations in this study were dysuria, urine urgency and urgency incontinence. The frequent UPEC virulence gene identified was fimH 164 (82%), followed by aer 109 (54.5%), hly 103 (51.5%), pap 59 (29.5%), cnf 58 (29%), sfa 50 (25%) and afa 24 (12%).There was significant association between pap gene and urine urgency (*p*-0.016); sfa and dysuria and urine urgency (*p*-0.019 and *p*-0.043 respectively); hly and suprapubic pain (*p*-0.002); aer and suprapubic pain, flank pain and fever (*p*-0.017, *p*-0.040, *p*-0.029 respectively). Majority of *E. coli* isolates were phylogroup B2 60(30%) followed by D 55(27.5%), B1 48(24%) and A 37(18.5%). There was significant association between *E. coli* phylogroup B2 and three virulence genes namely afa, pap, and sfa (*p*-0.014, *p*-0.002, *p*-0.004 respectively).

**Conclusion:**

In this study the most frequent *E. coli* virulence gene was fimH, followed by aer, hly, pap, cnf, sfa and afa respectively. There was significant association between *E. coli* virulence genes and clinical symptoms of UTI. The phylogenetic analysis indicates majority of uropathogenic *E. coli* isolates were phylogroup B2 followed by phylogroup D. Phylogroup B2 carries more virulence genes. Hence, targeting major UPEC phylogroup and virulence genes for potential vaccine candidates is essential for better management of UTI and further research has to be conducted in this area.

## Background

Urinary tract infection (UTI) remains the most common bacterial infection in human population and is also one of the most frequently occurring nosocomial infections [1]. Its annual global incidence is of almost 250 million [2–4]. *Escherichia coli* is the major etiologic agent in causing UTI, which accounts for up to 90% of cases [3]. *Escherichia coli* strains isolated from the urinary tract are known as uropathogenic *Escherichia coli* [5].

Uropathogenic *E. coli* strains possess an arsenal of virulence factors that contribute to their ability to overcome different defense mechanisms cause disease. These virulence factors that are located in virulence genes include fimbriae (which help bacterial adherence and invasion), iron-acquisition systems (which allow bacterial survival in the iron-limited environment of the urinary tract), flagella and toxins (which promote bacterial dissemination).Virulence genes are located on transmissible genetic elements (plasmid) and/or on the chromosome [6] so that non-pathogenic strains acquire new virulence factors from accessory DNA [7].

*Escherichia coli* strains derive from different phylogenetic groups; phylogenetic typing in four groups: A, B1, B2 and D. The majority of strains responsible for extraintestinal infections, including urinary tract infections, belong to group B2 or, to a lesser degree, to group D, whereas commensal isolates belong to groups A and B1 [8, 9]. To the best of our knowledge, there is no information on phylogenetics and genes that encode virulence factors of uropathogenic *E. coli* in Ethiopia. So, knowing the phylogroup and virulence factors of *E. coli* responsible for UTI is important for proper management, prevention and control of urinary tract infection.

## Methods

A cross sectional study was conducted from January 1, 2017 to October 9, 2017 in selected health facilities of Addis Ababa, Ethiopia; Namely Tikur Anbessa Specialized Hospital, Yekatit 12 Hospital and Zewditu memorial Hospital. These governmental hospitals were selected because they have microbiology laboratories that perform culture and antimicrobial sensitivity testing. They are also referral hospitals so most patients from Addis Ababa visit these hospitals. Clinical data were collected using a well-designed questionnaire.

The proposal of this study was ethically approved by the Institutional Review Board (IRB) of Addis Ababa University, College of Health Sciences. Permission was obtained from Medical directors of Tikur anbessa specialized Hospital, Yekatit 12 Hospital and Zewditu Hospital. Written informed consent was obtained from each patient participated in the study. Research participants were those patients coming to Tikur Anbessa Specialized Hospital, Zewditu Memorial Hospital and Yekatit 12 Hospital that were diagnosed with urinary tract infections and gave urine sample for microbiological investigation. The study participants’ age was > 1 year old (Those children age < 1 year were excluded from the study because it is difficult to obtain urine from these patients). Socio-demographic and clinical data were collected from the patient directly and from patient record respectively by data collectors using well designed questionnaire. Urine sample processing and microbiological investigations were conducted without delay in the Microbiology laboratory. Mid-stream urine sample was collected using sterile container from patients diagnosed with urinary tract infections. *Escherichia coli* isolates were presumptively identified by colonial morphology on MacConkey agar (Oxoid, UK), and further identified and confirmed by conventional biochemical tests. A sample was considered as positive for UTI if a single organism was cultured at a concentration of > 10^5^ CFU (colony forming unit) per milliliter of urine [10]. Patients having at least two of the following complaints: dysuria, urine urgency, frequency, incontinence, suprapubic pain, flank pain or costo-vertebral angle tenderness, fever (> 38 °C) and chills was considered as urinary tract infection.

In-vitro antimicrobial susceptibility testing of the bacterial isolates was performed by Kirby-Bauer disc diffusion method. The following antimicrobial agents were used with their respective concentration: trimethoprim-sulfamethoxazole (SXT) (1.25/23.75 μg), ampicillin (AMP) (10 μg), nalidixic acid (NA) (30 μg), amoxicillin-clavulanate (AMC) (20/10 μg), ceftazidime (CAZ) (30 μg), tetracycline (TE) (30 μg), cefotaxime (CTX) (30 μg), ceftriaxone (CRO) (30 μg), gentamicin (CN) (10 μg), ciprofloxacin (CIP) (5 μg), amikacin (AK) (30 μg), norfloxacin (NOR) (10 μg), nitrofurantoin (F) (300 μg), meropenem (MEM) (10 μg), imipenem (IM) (10 μg) and chloramphenicol (C) (30 μg) (Oxoid, UK). The antibiotic disks were firmly placed on sterile Mueller-Hinton Agar (Oxoid, UK) plates previously seeded with a 24 h old culture of the isolate (10^6^ CFU/ml of 0.5 McFarland Standard). The plates were incubated at 37 °C for 24 h and diameter of zones of inhibitions was measured using caliper and compared with the standard set by CLSI [11]. *E. coli* ATCC 25922 was used as reference strain. Molecular characterization of *E. coli* isolates was conducted in college of Human Medicine, Michigan State University, USA.

### Bacterial DNA extraction

DNA extraction was performed using an alkaline heat lysis method. *Escherichia coli* strains were grown on LB medium at 37 °C overnight. Bacteria colonies were inoculated and suspended in 1.5 ml centrifuge tubes containing 200 μl of 1xPBS solution, and then 800 μl of 0.05 M NaOH added and mixed by vortexing. The sample/mixture was incubated at 60 °C for 45 min. After 45 min 240 μl 1 M Tris-Cl was added to neutralize NaOH and centrifuged at 13,000 rpm for 3 min. One thousand microliters of the supernatant were stored at -20 °C as a template DNA stock [12, 13].

### Detection of virulence genes of uropathogenic *Escherichia coli*

The genetic determinants that are studied includes those coding for type 1 fimbriae [*fimH*], pili associated with pyelonephritis [*pap*], S and F1C fimbriae [*sfa* and *foc*], afimbrial adhesins [*afa*], hemolysin [*hly*], cytotoxic necrotizing factor [*cnf*], and aerobactin [*aer*] [12, 14–16].Specific primers were used to amplify sequences of the *fim*, *pap*, *sfa/foc*, *afa*, *hly*, *cnf*, and *aer* operons. Details of primer sequences and predicted sizes of the amplified products are given in Table 1.

**Table 1:** Primers for uropathogenic *Escherichia coli* virulence genes PCR assay [12, 16]

Virulence factor	Target gene(s)	Primer Name	Primer Sequence (5′- 3′)	Size of amplicon (bp)
Type 1 fimbriae	*fimH*	fimH-f	5′-AACAGCGATGATTTCCAGTTTGTGTG-3′	465
		fimH-r	5′-ATTGCGTACCAGCATTAGCAATGTCC-3′	
P fimbriae	*papC*	pap1	5′-GACGGCTGTACTGCAGGGTGTGGCG-3’	328
		pap2	5′-ATATCCTTTCTGCAGGGATGCAATA-3’	
S and FIC fimbriae	*Sfa/focDE*^*h*^ *region*	sfa1	5′-CTCCGGAGAACTGGGTGCATCTTAC-3’	410
		sfa2	5′-CGGAGGAGTAATTACAAACCTGGCA-3’	
Afa adhesins	*afaC*^*c*^	afa-f	5′-CGGCTTTTCTGCTGAACTGGCAGGC-3’	672
		afa-r	5′-CCGTCAGCCCCCACGGCAGACC-3’	
Hemolysin	*hlyCA region*	hly s	5′-AGATTCTTGGGCATGTATCCT-3’	556
		hly as	5′-TTGCTTTGCAGACTGTAGTGT-3’	
Cytotoxic necrotizing factor	*cnf*	cnf s	5′-TTATATAGTCGTCAAGATGGA-3’	693
		cnf as	5′-CACTAAGCTTTACAATATTGA-3’	
Aerobactin	*iucC*	aer s	5′-AAACCTGGCTTACGCAACTGT-3’	269
		aer as	5′-ACCCGTCTGCAAATCATGGAT-3’	

Detection of *fim*, *pap* and *afa,* and *sfa/foc* and *aer* sequences were done by multiplex PCR while *hly* and *cnf* detection were done by single-plex PCR [12, 16, 17]. PCR amplification of bacterial DNA extracts was done in a total volume of 25 μl containing 20 μl of Platinum® PCR SuperMix (The mixture contains Mg^++^, dNTPs and recombinant *Taq* DNA polymerase at concentrations sufficient to allow amplification during PCR), 1.5 μl template DNA and1.5–2 μl (30 pmol of each) of the primers [12, 16].

The amplification was carried out in a multiplex PCR [T100™ Thermal cycler (BIO RAD) & PTC-200 Peltier Thermal cycler (MJ Research)]. Conditions consisted of an initial denaturation at 94 °C for 10 min, followed by 30 cycles of denaturation at 94 °C for 2 min, annealing at a specific temperature for 30 s (Multiplex PCR for *fimH, afa* and *pap* annealing temperature used was 60 °C; Multiplex PCR for *sfa* and *aer* annealing temperature used was 55 °C; annealing temperature of Single-plex PCR for *cnf* and *hly* was 45 °C and 50 °C respectively) and 72 °C for 1 min, and final extension at 72 °C for 10 min. A 4.5 μl aliquot of the PCR product was mixed with 6x blue loading dye on parafilm and loaded on 1.2% agarose gel electrophoresis stained with 10 μL 10,000x GelRed. Electrophoresis was carried out for 120 min at 110 V on TAE buffer system and the gel was imaged under UV light (E-gel Imager; life technologies, USA). Amplified DNA fragments of specific sizes were detected by UV-induced fluorescence and the size of the amplicons were estimated by comparing them with the 1 kb plus DNA ladder (Invitrogen™) included on the same gel [12, 16].

Strain J96 was used as positive control for *pap, sfa/foc, hly, cnf,* and *fimH* sequences and the strain K10 was used as positive control for *afa*. The positive control for *aer* was J96 and Cl_1212_strains and distilled water is used as negative control [18–20].

### Phylogenetics grouping of uropathogenic *Escherichia coli*

*E. coli* strains responsible for extra-intestinal infection are far more likely to be members of phylogroups B2 or D than A or B1 [7, 8, 21]. This study used PCR assay to detect the genes *chuA* and *yjaA,* and an anonymous DNA fragment *TspE4.C2* found in *E. coli* isolates to classify *E. coli* isolates into phylogroups A, B1, B2 or D [22] (See Table 2). All PCR reactions were carried out in a 25 μl volume containing 20 μl of 10X buffer (supplied with Taq polymerase), 2 mM each dNTP, 2 U of Taqpolymerase (Invitrogen™ Super mix); the amounts of primer used are 20 pmol (2 μl of each primers). PCR reactions (T100™ Thermal cycler, BIO RAD) were performed under the following conditions: denaturation 4 min at 94 °C, 30 cycles of 5 s at 94 °C and 20 s at 59 °C, and a final extension step of 5 min at 72 °C [23, 24]. Interpretation of amplified PCR products for phylogrouping *E. coli* was done according to Clermont et al. [22] (See Table 3).

**Table 2:** Primers used for phylogenetic of uropathogenic *Escherichia coli* [23]

Primer Name	Gene Target	Nucleotide Sequence	PCR Product (bp)
chuA.1b	*chuA*	5′-ATGGTACCGGACGAACCAAC-3′	288
chuA.2		5′-TGCCGCCACTACCAAAGACA-3′	
yjaA.1b	*yjaA*	5′-CAAACGTGAAGTGTCAGGAG-3′	211
yjaA.2b		5′-AATGCGTTCCTCAACCTGTG-3′	
TspE4C2.1b	*TspE4.C2*	5′-CACTATTCGTAAGGTCATCC-3′	152
TspE4C2.2b		5′-AGTTTATCGCTGCGGGTCGC-3′	
AceK.f	*arpA*	5′-AACGCTATTCGCCAGCTTGC-3’	400
ArpA1.r		5′-TCTCCCCATACCGTACGCTA-3’

**Table 3:** Interpretation of amplified PCR products for phylogrouping *E. coli* [22]

Phylogroup	*chua*	*yjaA*	*TSPEA.C2*
A	–	–	–
A	–	+	–
B1	–	–	+
B1	–	+	+
B2	+	+	+
B2	+	+	–
D	+	–	–
D	+	–	+

### Data analysis

SPSS version 16.0 and Epi-info version 3.4.1 softwares were used for data analysis. Regression and Chi-square test was performed to asses’ relationship between variables. *P* value < 0.05 was considered as significant.

## Results

Urine samples of 780 study participants who had complaints of urologic symptoms of urinary tract infections were cultured and 200 (25.6%) *Escherichia coli* isolates were identified by biochemical tests. Among study participants, 265 (34%) were males and 515 (66%) were females.

The most common urologic clinical manifestation combinations in this study were dysuria; urine urgency and urgency incontinence followed by dysuria and urgency incontinence (see Table 4).

**Table 4:** Common combinations of clinical manifestations

Clinical data combinations	Frequency
Dysuria, urine urgency, urgency incontinence	176
Dysuria, urgency incontinence	100
Dysuria, urine urgency, flank pain	80
Dysuria, urine urgency	78
Dysuria, urgency incontinence, suprapubic pain	60
Urine urgency, urgency incontinence	54
Dysuria, suprapubic pain, flank pain	50
Dysuria, suprapubic pain	42

The antimicrobial susceptibility patterns of 200 *E. coli* isolates which were subjected to similar testing procedure showed that *E. coli* isolates had highest resistance (86.5%) to ampicillin followed by ceftazidime (84%), ceftriaxone (80.5%), tetracycline (80%), trimethoprim-sulfamethoxazole (68.5%) and cefotaxime (66%) (see Table 5).

**Table 5:** Antimicrobial susceptibility patterns of *E. coli* isolates

Antimicrobial agents	Number of resistance (%)	Number of intermediate (%)	Number of susceptible (%)
Ciprofloxacin	29 (14.5)	0	171 (85.5)
Norfloxacin	30 (15)	0	170 (85)
Nitrofurantoin	10 (5)	0	190 (95)
Trimethoprim-sulfamethoxazole	137 (68.5)	0	63 (31.5)
Tetracycline	160 (80)	0	40 (20)
Ceftriaxone	161 (80.5)	9 (4.5)	30 (15)
Ampicillin	173 (86.5)	6 (3)	21 (10.5)
Nalidixic acid	42 (21)	0	158 (79)
Amoxicillin-clavulanate	58 (29)	36 (18)	106 (53)
Ceftazidime	168 (84)	19 (9.5)	13 (6.5)
Cefotaxime	132 (66)	2 (1)	66 (33)
Amikacin	5 (2.5)	0	195 (97.5)
Meropenem	0	0	200 (100)
Imipenem	0	0	200 (100)
Chloramphenicol	33 (16.5)	0	167 (83.5)
Gentamicin	40 (20)	0	160 (80)

**Fig. 1:**
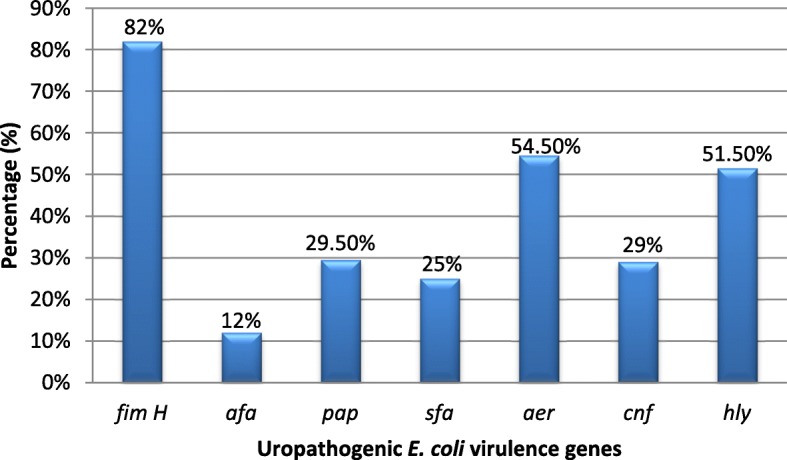
Virulence genes of uropathogenic *Escherichia coli*. (Proportion of uropathogenic *E. coli* virulence genes. The most frequent virulence gene was *fim H*, followed by *aer*, *hly*, *pap*, *cnf*, *sfa* and *afa*)

Virulence genes were amplified and detected successfully in 198 (99%) *E. coli* isolates. The most frequent *E. coli* virulence gene was *fimH* 164 (82%), followed by *aer* 109 (54.5%), *hly* 103 (51.5%), *pap* 59 (29.5%), *cnf* 58 (29%), *sfa* 50 (25%) and *afa* 24 (12%) (see Fig. 1).

**Fig. 2:**
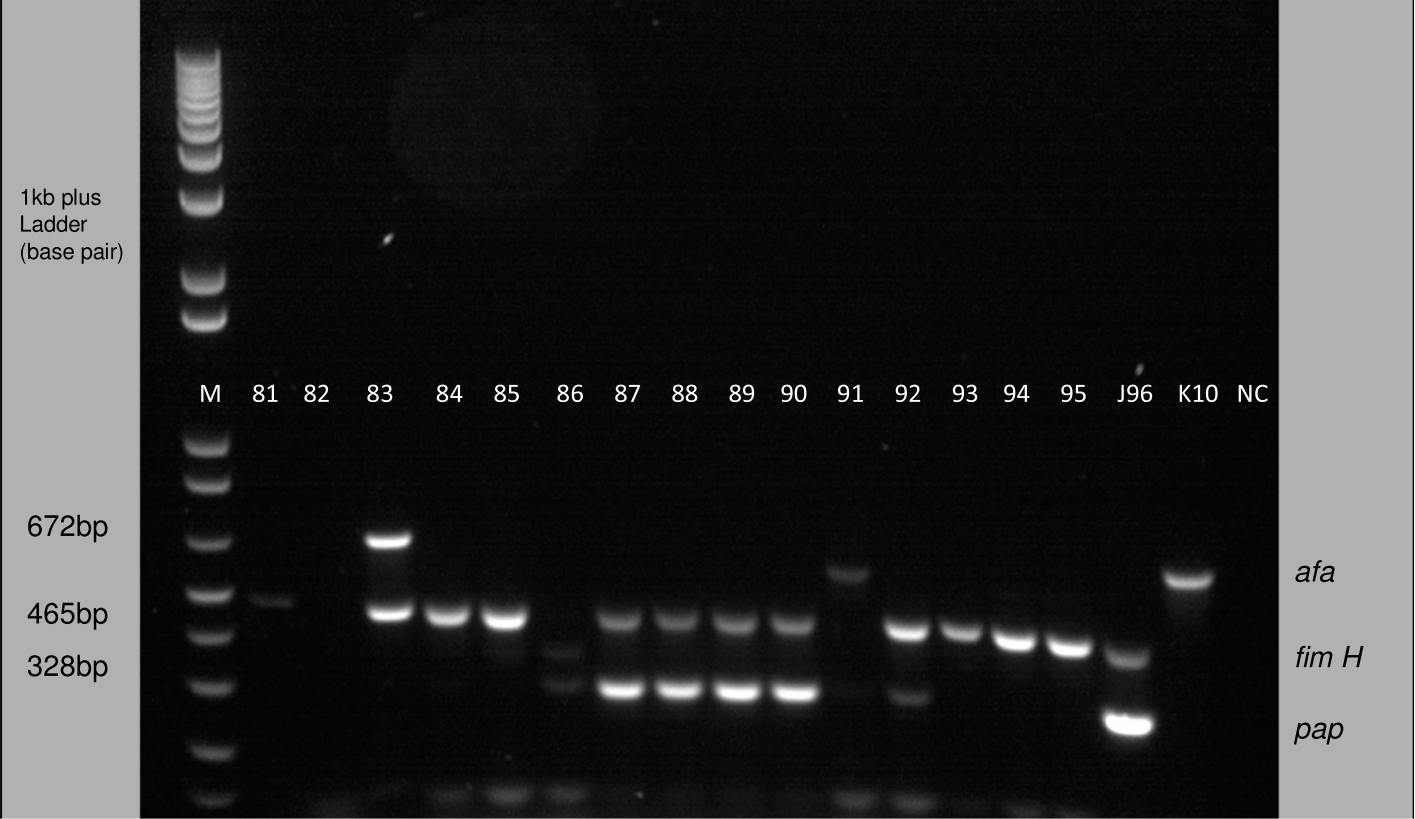
Representative 1.2% agarose gel electrophoresis of uropathogenic *Escherichia coli* virulence genes isolated from urinary tract infection patients. (Lane M, 1-kb plus ladder; lane 1–15, amplified *E. coli* PCR products (sample 81–95) with the following virulence genes, *pap* (328 bp), *fimH* (456 bp) and *afa* (672 bp); lane 16, strain J96 positive control; lane 17, strain K10 positive control and lane 20, distilled water as negative control)

The virulence genes *fimH* (456 bp), *afa* (672 bp), *pap* (328 bp), *sfa* (410 bp), *aer* (269 bp), *cnf* (693 bp) and *hly* (556 bp) were successfully amplified. One kilobase plus (1 kb plus) DNA ladder was used to determine the base pair size (see Fig. 2).

There was significant association between *pap* gene and urine urgency (*p-0.016*); *sfa* and dysuria and urine urgency (*p*-0.019 and *p*-0.043 respectively); *hly* and suprapubic pain (*p*-0.002); *aer* and suprapubic pain, flank pain and fever (*p*-0.017, *p*-0.040, *p*-0.029 respectively) (see Table 6).

**Table 6:** Association between virulence genes of *E. coli* and clinical data of urinary tract infections

Clinical data		Virulence genes															
		*fim H*				*afa*				*pap*				*sfa*			
		Present	Absent	OR (95% CI)	*P*-value	Present	Absent	OR (95% CI)	*P*-value	Present	Absent	OR (95% CI)	*P*-value	Present	Absent	OR (95% CI)	*P*-value
Dysuria	Present	146	29	1.958 (0.750,5.112)	0.170	20	155	0.677 (0.211,2.174)	0.512	51	124	0.874 (0.355,2.153)	0.770	39	136	0.365 (0.153,0.868)	0.019
	Absent	18	7			4	21			8	17			11	14		
Urine urgency	Present	116	30	0.483 (0.189,1.236)	0.123	15	131	0.573 (0.234,1.398)	0.217	50	96	2.604 (1.178,5.756)	0.016	42	104	2.322 (1.011,5.336)	0.043
	Absent	48	6			9	45			9	45			8	46		
Urgency incontinence	Present	95	27	0.459 (0.203,1.037)	0.057	12	110	0.600 (0.255,1.413)	0.239	36	86	1.001 (0.537,1.867)	0.997	31	91	1.058 (0.548,2.043)	0.867
	Absent	69	9			12	66			23	55			19	59		
Suprapubic pain	Present	73	12	1.604 (0.752,3.425)	0.219	9	76	0.789 (0.328,1.901)	0.597	20	65	0.600 (0.319,1.129)	0.111	23	62	1.209 (0.635,2.302)	0.563
	Absent	91	24			15	100			39	76			27	88		
Flank pain	Present	88	17	1.294 (0.628,2.666)	0.484	10	95	0.609 (0.257,1.445)	0.257	28	77	0.751 (0.408,1.380)	0.356	31	74	1.676 (0.871,3.225)	0.120
	Absent	76	19			14	81			31	64			19	76		
Fever	Present	22	4	1.239 (0.399,3.846)	0.479	3	23	0.950 (0.262,3.441)	0.619	8	18	1.072 (0.438,2.622)	0.879	6	20	0.886 (0.335,2.348)	0.808
	Absent	142	32			21	153			51	123			44	130		
Chills	Present	10	1	2.273 (0.282,18.341)	0.693	1	10	0.722 (0.088,5.902)	0.611	4	7	1.392 (0.392,4.947)	0.735	3	8	1.133 (0.289,4.447)	0.549
	Absent	154	35			23	166			55	134			47	142		
		*aer*				*cnf*				*hly*							
		Present	Absent	OR (95% CI)	*P*-value	Present	Absent	OR (95% CI)	*P*-value	Present	Absent	OR (95% CI)	*P*-value				
Dysuria	Present	96	79	1.122(0.485,2.596)	0.788	48	127	0.567(0.238, 1.348)	0.195	92	83	1.411(0.607, 3.280)	0.422				
	Absent	13	12			10	15			11	14						
Urine urgency	Present	78	68	0.851(0.453,1.598)	0.616	46	100	1.610(0.776, 3.342)	0.199	79	67	1.474(0.787, 2.761)	0.225				
	Absent	31	23			12	42			24	30						
Urgency incontinence	Present	71	51	1.465(0.828,2.595)	0.189	38	84	1.312(0.694, 2.479)	0.403	65	57	1.200(0.680, 2.120)	0.529				
	Absent	38	40			20	58			38	40						
Suprapubic pain	Present	38	47	0.501(0.284,0.885)	0.017	28	57	1.392(0.753, 2.574)	0.291	33	52	0.408(0.230, 0.725)	0.002				
	Absent	71	44			30	85			70	45						
Flank pain	Present	50	55	0.555(0.315,0.975)	0.040	33	72	1.283(0.694, 2.374)	0.426	53	52	0.917(0.526,1.599)	0.761				
	Absent	59	36			25	70			50	45						
Fever	Present	9	17	0.392(0.165,0.928)	0.029	11	15	1.982(0.850, 4.622)	0.109	13	13	0.933(0.409,2.128)	0.870				
	Absent	100	74			47	127			90	84						
Chills	Present	4	7	0.457(0.129,1.614)	0.214	3	8	0.914(0.234, 3.572)	0.600	4	7	0.519(0.147,1.834)	0.301				
	Absent	105	84			55	134			99	90						

### Phylogenetics of uropathogenic *Escherichia coli*

**Fig. 3:**
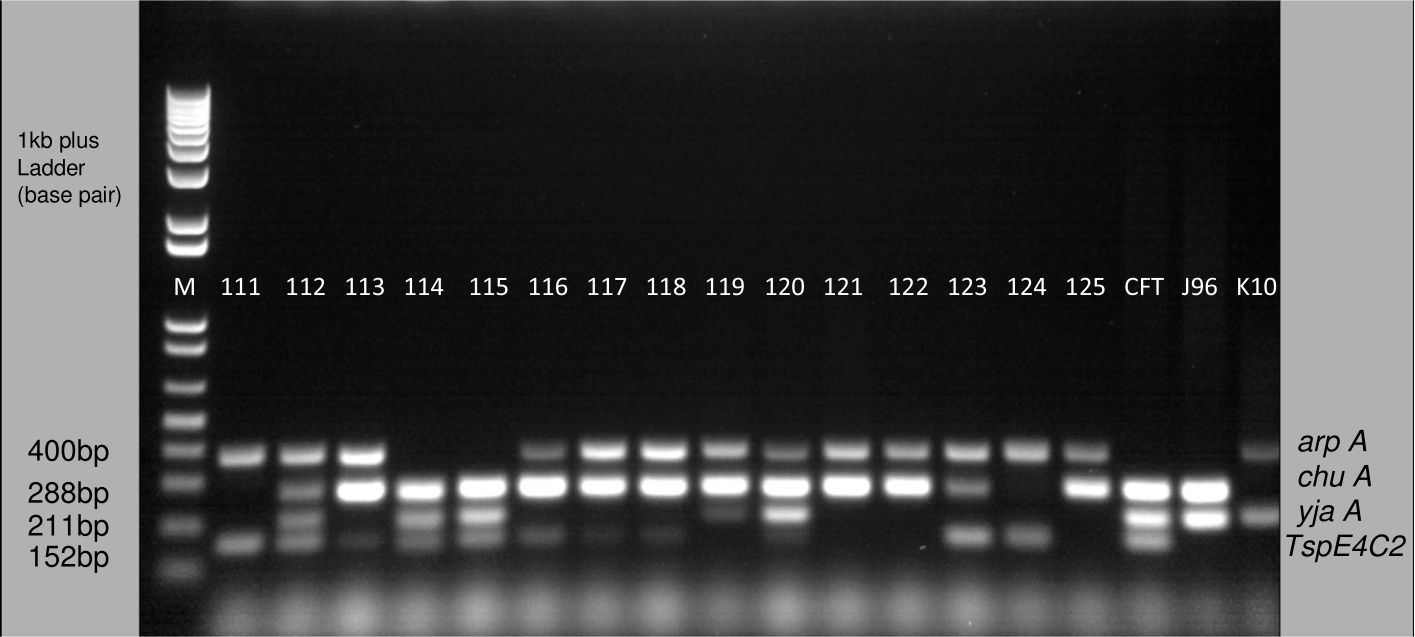
Representative 1.2% agarose gel electrophoresis of uropathogenic *Escherichia coli* genes used to classify *Escherichia coli* into different phylogroup. (Lane M, 1-kb plus ladder; lane 1–15, amplified PCR products (sample 111–125) with the following *Escherichia coli* phylogrouping genes; *arp A* (400 bp), *chu A* (288 bp), *yja A* (211 bp) and *TspE4C2* (152 bp); lane 16, strain CFT073 positive control; lane 17, strain J96 positive control and lane 20, strain K10 positive control)

The distribution of phylogenetic groups amongst *Escherichia coli* isolates was determined by the following genes; *arp A* (400 bp), *chu A* (288 bp), *yja A* (211 bp) and an anonymous DNA fragment that is found in *E. coli* worldwide; *TspE4C2* (152 bp). One kilobase plus (1 kb plus) DNA ladder was used to determine the base pair size (see Fig. 3).

**Fig. 4:**
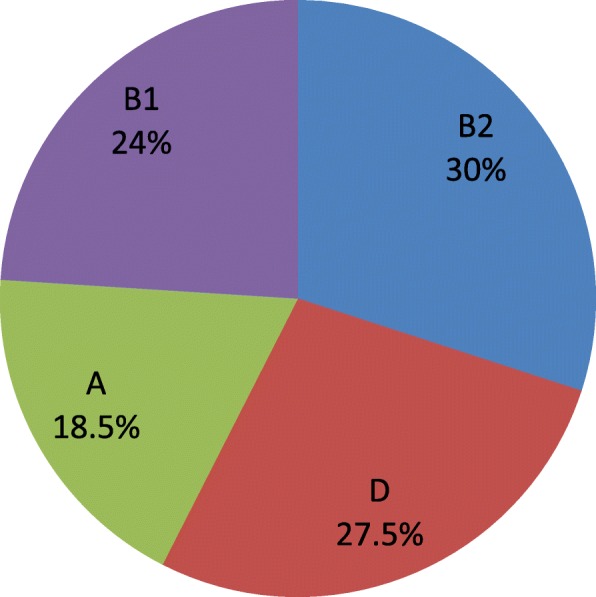
Phylogroup distribution of uropathogenic *Escherichia coli* isolates. (The most common phylogroup of uropathogenic *Escherichia coli* isolates was phylogroup B2 followed by phylogroup D, phylogroup B1 and phylogroup A)

Phylogenetic analysis indicates majority of uropathogenic *Escherichia coli* isolates were group B2 60(30%) followed by group D 55(27.5%), group B1 48(24%) and group A 37(18.5%) (see Fig. 4).

In this study there was significant association between *Escherichia coli* phylogroup B2 and three virulence genes namely *afa*, *pap*, and *sfa* (*p*-0.014, *p*-0.002, *p*-0.004 respectively). Similarly, there was significant association between *Escherichia coli* phylogroup D and two virulence genes namely *fimH* and *pap* (*p*-0.043, *p*-0.019 respectively). There was significant association between *Escherichia coli* phylogroup A and virulence genes *fimH* and *afa* (*p*-0.011, *p*-0.002 respectively). Phylogroup B1 has significant association with *pap* gene (*p*-0.001). The virulence factor that encodes *pap* gene has significant association with *Escherichia coli* phylogroup B2, D and B1 (*p*-0.002, *p*-0.019, *p*-0.001 respectively) (see Table 7).

**Table 7:** Association between phylogroup and virulence genes of uropathogenic *Escherichia coli*

Virulence genes		Phylogroup															
		A				B1				B2				D			
		Present	Absent	X^2^	*P*-value	Present	Absent	X^2^	*P*-value	Present	Absent	X^2^	*P*-value	Present	Absent	X^2^	*P*-value
*fim H*	Present	25	139	6.407	0.011	41	123	0.500	0.480	48	116	0.232	0.630	50	114	4.079	0.043
	Absent	12	24			7	29			12	24			55	31		
*afa*	Present	10	14	9.708	0.002	5	19	0.150	0.699	2	22	6.097	0.014	7	17	0.038	0.845
	Absent	27	149			43	133			58	118			48	128		
*pap*	Present	6	53	3.852	0.050	3	56	16.416	0.001	27	32	9.902	0.002	23	36	5.535	0.019
	Absent	31	110			45	96			33	108			32	109		
*sfa*	Present	7	43	0.895	0.344	9	41	1.316	0.251	23	27	8.127	0.004	11	39	1.011	0.315
	Absent	30	120			39	111			37	113			44	106		
*aer*	Present	25	84	3.126	0.077	22	87	1.913	0.167	38	71	2.697	0.101	24	85	3.610	0.057
	Absent	12	79			26	65			22	69			31	60		
*cnf*	Present	9	49	0.482	0.488	13	45	0.113	0.737	23	35	3.626	0.057	13	45	1.060	0.303
	Absent	28	114			35	107			37	105			42	100		
*hly*	Present	17	86	0.561	0.454	25	78	0.009	0.926	36	67	2.479	0.115	25	78	1.110	0.292
	Absent	20	77			23	74			24	73			30	67		

## Discussion

In this study higher proportion of urinary tract infections in females (66%) than in males (34%) were observed. UTI is more common in females than in males because structurally the female urethra is less effective in preventing the bacterial entry for colonization i.e. the urethra is shorter and wider. *Escherichia coli* is common because it is a normal flora in large intestine and can easily be acquired via faecal contamination with urinary tract especially in female it causes ascending UTI [25]. The highest incidence of urinary tract infections was observed in the age groups 26–45. This could be due to the fact that this age group is sexually active. Sexual intercourse may access entry of bacteria in to bladder. Identification of virulence factors that are encoded by uropathogenic *E. coli* are important for pathogenesis, severity of urinary tract infection, targets for vaccine and drug development [26].

In our study *fimH* adhesion gene was the most common and present in 164 (82%) uropathogenic *E. coli* isolates which is in agreement with studies conducted in Romania, 86% [16]; Mongolia, 89.9% [27], Iran, 86.17% [28], 79.67% [29] and China, 87.4% [30]. Targeting *fimH* as vaccine candidate is important for prevention of UTI and currently vaccine targeting *fimH* as potential vaccine candidate is under investigation. Antibodies against *fimH* prevent colonization of urinary tract by UPEC isolates [26].In this study, we found no significant association between *fimH* gene and clinical symptoms of UTI (*p* > 0.05), but this does not mean *fimH* is not involved in pathogenesis of UTI.

Pyelonephritis associated pili (*pap*) gene was found in 59 (29.5%) uropathogenic *E. coli* isolates which is comparable to study conducted in Iran, 30.2% [6]; Mexico, 24.7% [31]; Romania, 36% [16] and Brazil, 32% [32]; but lower than studies conducted in Iran, 50.4% [28], 57% [33] and Egypt 54% [34]. In this study, there was significant association between presence of *pap* gene and urine urgency (*p-0.016*), which was commonly observed clinical symptom in most UTI patients. This indicates that UPEC uses *pap* genes as virulence factor to cause UTIs.

S and F1C fimbriae (*sfa* gene) was found in 50 (25%) uropathogenic *E. coli* isolates which is similar to studies conducted in Pakistan, 27% [35]; Romania, 23% [16]; Tunisia, 34% [12]; Iran, 32% [36] and Iraq, 22.7% [37]; but lower than studies conducted in Denmark, 46% [38]; Iran, 81% [33] and South Korea, 100% [39] and higher than studies conducted in Mongolia, 8.8% [27] and China, 8% [30]. In our study, there was significant association between presence of *sfa* gene, and dysuria and urine urgency (*p*-0.019 and *p*-0.043 respectively). This indicates that *sfa* genes are important for pathogenesis of UPEC to cause UTI and responsible for clinical symptoms of UTIs.

Afa adhesin (*afa* gene) was found in 24 (12%) uropathogenic *E. coli* isolates which is similar to studies conducted in Iran, 12% [33]; Mexico, 12.8% [31]; Brazil, 11% [32] and Romania, 14% [16]. Afimbrial adhesins (*afa*) may favor establishment of chronic and/or recurrent urinary tract infections [40]. In this study, we found no significant association between *afa* gene and clinical symptoms of UTI (*p* > 0.05).

Uropathogenic *E. coli* secretes toxins like α-haemolysin (*hlyA*) and cytotoxic necrotizing factor 1 (*cnf1*). *Hly* Alpha promotes bladder cell exfoliation and cell lysis, which facilitates iron and nutrient acquisition by the bacteria. *Cnf1*involved in bladder cell exfoliation and increased levels of bacterial internalization [26, 41].

In this study 103 (51.5%) uropathogenic *E. coli* isolates carries hemolysin (*hly*) gene which is comparable to studies conducted in Iran, 50.4% [28] and South Korea, 62% [39]; but higher than studies conducted in Zimbabwe, 12.5% [14]; Tunisia, 19% [12]; Poland, 18.5% [42]; Mexico, 15.4% [31] and China, 11.6% [30].In our study, hemolysin gene was significantly associated with suprapubic pain (*p*-0.002). This indicates that hemolysin may be responsible for clinical manifestation in UTI patients. Alpha-hemolysin encoded by *hlyA* is an extracellular cytolytic protein toxin that is produced by up to 50% of UPEC isolates. Alpha-hemolysin has been associated with clinical severity in UTI patients [43]. Currently vaccine against *hlyA* that protect renal damage is under investigation [44].

In our study we found 58 (29%) uropathogenic *E. coli* isolates carries cytotoxic necrotizing factor 1 (*cnf1*) which is similar to studies conducted in Iran, 36.5% [45] and Pakistan, 20% [35]; but higher than studies conducted in Tunisia, 3% [12]; Romania, 13% [16] and Poland, 12.1% [42]. In this study, we found no significant association between *cnf1* gene and clinical symptoms of UTI (*p* > 0.05).

Iron is generally required for bacterial growth during infection. Thus UPEC stains uses iron acquisition genes like aerobactin, *aer* [46]. In this study we found 109 (54.5%) uropathogenic *E. coli* isolates carries aerobactin (*aer*) genes which is similar to studies conducted in Romania, 54% [16]; Tunisia, 52% [12]; Egypt, 51% [34] and Poland, 52.6% [42]; but lower than studies conducted in South Korea, 81% [39] and Iran, 73.1% [45]. Currently, Siderophore proteins are under investigation for potential vaccine candidate against UTI [26].There was significant association between *aer* gene and suprapubic pain, flank pain and fever (*p*-0.017, *p*-0.040, *p*-0.029 respectively). Thus, the high prevalence of *aer* gene in our study may be due to UPEC utilizes aerobactin virulence gene as a means of acquisition of iron and associated with clinical features of suprapubic pain, flank pain and fever which were observed in most UTI patients.

From clinical point of view UTI is classified as upper (proximal) urinary tract infection and lower (distal) urinary tract infection. Upper urinary tract infection includes pyelonephritis while lower urinary tract infection includes cystitis and urethritis. The common clinical symptom of upper urinary includes flank pain, fever, chills and costo-vertebral angle tenderness. The common clinical symptom of lower urinary includes dysuria and frequency. The above mentioned clinical symptoms of UTI that is correlated with virulence genes should be interpreted in this aspect.

The difference in virulence genes prevalence between our study and different studies abroad may be due to sample size difference and methodology difference. Several virulence determinants are the product of different genes, which can be detected by PCR method [16, 28, 42, 45]. However, when there is mutation at the level of the corresponding gene, this will lead to negative PCR. Thus negative PCR doesn’t mean absence of specific virulence gene [12].

Phylogenetic analyses have shown that virulent uropathogenic *E. coli* strains belonged typically to group B2 and less often to group D [38, 39]. Our finding is in agreement with studies conducted in Denmark [38], Pakistan [35], South Korea [39], Poland [42] and Mexico [31] where it was found that the majority of isolates of *E. coli* predominantly belong to phylogenetic group B2. These findings are indicative of virulent strains of UPEC are common in study area among UTI patients and measures needs to be taken to combat these virulence strains through designing and implementing appropriate prevention and control strategies.

In our study the phylogenetic analysis indicated majority of uropathogenic *E. coli* isolates were group B2 60(30%) followed by group D 55(27.5%), group B1 48(24%) and group A 37(18.5%) which is in agreement with study conducted by Munkhdelger et al [27], where B2 (33.8%) was dominant strains followed by D (28.4%) strains, A (19.6%) strains and B1 (18.2%) strains. Similar study conducted by Kot et al [42], showed that 38.1% *E. coli* strains belonged to phylogenetic group B2, 35.3% to group D, 18.5% to group A, and 8.1% to group B1. Phylogenetic group A, represented 18% of isolates, which was higher than in studies conducted in South Korea 3.44% [39] and Iran 0.7% [47], but some studies found phylogroup A was the dominant phylogroup [9, 15, 48] suggesting that the colon may be the main reservoir for strains that cause urinary tract infections [9]. In some studies, phylogroup D was the dominant strain [25, 49]. These different prevalence of the phylogenetic groups may be due to health status of the host, and geographic conditions, or variations in methodology and sample size [50].

In our study there was significant association between *E. coli* phylogroup B2 and three virulence genes namely *afa*, *pap*, and *sfa* (*p*-0.014, *p*-0.002, *p*-0.004 respectively). This finding is explained by the fact that *E. coli* strains belonging to phylogroup B2 contained a greater number of virulence genes than *E. coli* than other phylogroup as reported by other studies on UPEC isolates [38, 51]. There was also significant association between *E. coli* phylogroup D and two virulence genes namely *fimH* and *pap* (*p*-0.043, *p*-0.019 respectively) which is in agreement with a study conducted in Thailand [25].

In this study high prevalence of drug resistance to ceftazidime (84%), ceftriaxone (80.5%), and cefotaxime (66%) was observed [52]. Resistance to ceftriaxone was observed in 80.5% of UPEC isolates, which is comparable to studies conducted in Nigeria 86% [13], India 81.8% [53] and China 84.8% [30], but higher than studies conducted in Nigeria 23.3% [54] and Mexico 10.2% [21]. Resistance to cefotaxime was observed in 66% of UPEC isolates, which is in agreement with studies conducted in Nigeria 68% [13], India 66.66% [55] and Iraq 78% [37], but higher than studies conducted in Ethiopia 18.7% [56], Nigeria 4.4% [9] and South Korea 7.8% [43]. Ceftriaxone is recommended for treatment of severe pyelonephritis in Ethiopia by national standard treatment guidelines [57]. Resistance to ceftriaxone could be due to transmission of resistant strains/isolates among hospitalized patients and noncompliance with medication. To reduce the incidence of resistance, empirical antibiotic selection in treatment of UTI must be based on the knowledge of local prevalence of causative uropathogens and their respective antimicrobial sensitivities rather than on universal guidelines [58].

### Limitations of the study

The triplex PCR phylogroup assignment used in this study has limitations like many strains could be potentially mis-assigned as this method has low sensitivity and expected to fail in allocating recombinant variants. Exclusion of infants < 1 years and not differentiating nosocomial and community acquired infections are the limitation of this study. Whole Genome Sequencing, Pulse Field Gel Electrophoresis and MLVA were not done.

## Conclusions

In our study the most frequent uropathogenic *E. coli* virulence gene was *fimH*, followed by *aer*, *hly*, *pap*, *cnf*, *sfa* and *afa* respectively. The most common urologic clinical manifestation combinations in this study were dysuria, urine urgency and urgency incontinence. There was significant association between *pap* gene and urine urgency; *sfa* and dysuria and urine urgency; *hly* and suprapubic pain; *aer* and suprapubic pain, flank pain and fever. The phylogenetic analysis indicates majority of uropathogenic *E. coli* isolates were phylogroup B2 followed by phylogroup D. There was significant association between *E. coli* phylogroup B2 and three virulence genes namely *afa*, *pap*, and *sfa*. Hence, targeting major uropathogenic *Escherichia coli* phylogroup and virulence genes for potential vaccine candidates is essential for better management of UTI and further research has to be conducted in this area.

## Data Availability

The datasets generated and/or analyzed during the current study are not publicly available due to ethical and confidentiality reasons but are available from the corresponding author on reasonable request under the Ethics Committee’s approval.

## Abbreviations

*aer*: aerobactin
*afa*: afimbrial adhesins
bp: base pair
CFU: Colony forming unit
CLSI: Clinical and Laboratory Standards Institute
*cnf*: cytotoxic necrotizing factor
DNA: Deoxyribonucleic acid
dNTPs: deoxynucleoside triphosphates
*fimH*: type1 fimbriae
*hly*: hemolysin
IRB: Institutional Review Board
NaOH: Sodium Hydroxide
*pap*: pili associated with pyelonephritis
PCR: Polymerase chain reaction
*sfa*: S and F1C fimbriae
UPEC: uropathogenic *E. coli*
UTI: Urinary tract infection

## References

[1] Gastmeir P, Kampf G, Wischnewski N, Hauer T, Schulgen G, Schumacher M (1998). Prevalence of nosocomial infections in representative German hospitals. J Hosp Infect.

[2] Ronald AR, Nicolle LE, Stamm E, Krieger J, Warren J, Schaeffer A (2001). Urinary tract infection in adults: research priorities and strategies. Int J Antimicrob Agents.

[3] Loh KY, Sivalingam N (2007). Urinary tract infections in pregnancy. Malaysian Fam Physician.

[4] Griebling TL (2005). Urologic diseases in American project: trends in resource use for urinary tract infections in women. J Urology.

[5] Schwartz DJ, Kalas V, Pinkner JS, Chen SL, Spaulding CN, Dodson KW (2013). Positively selected FimH residues enhance virulence during urinary tract infection by altering FimH conformation. Proc Natl Acad Sci U S A.

[6] Farshad S, Ranjbar R, Japoni A, Hosseini M, Anvarinejad M, Mohammadzadegan R (2012). Microbial susceptibility, virulence factors, and plasmid profiles of uropathogenic *Escherichia coli* strains isolated from children in Jahrom, Iran. Arch Iran Med.

[7] Johnson JR, Kuskowski MA, Obryan T, Colodner R, Raz R (2006). Virulence genotype and phylogenetic origin in relation to antibiotic resistance profile among *Escherichia coli* urine sample isolates from Israeli women with acute uncomplicated cystitis. Antimicrob Agents Chemother.

[8] Horcajada JP, Soto S, Gajewski A, Smithson A, Jiménez de Anta MT, Mensa J (2005). Quinolone-resistant uropathogenic *Escherichia coli* strains from phylogenetic group B2 have fewer virulence factors than their susceptible counterparts. J Clin Microbiol.

[9] Romanus II, Eze AT (2011). Antibiotics susceptibility patterns and clonal relatedness of uropathogenic *Escherichia coli* in Abakaliki, Ebonyi state. Canad J Pure Appl Sci.

[10] Farmer JJ. Enterobacteriaceae: introduction and identification. In: Murray PR, Baron EJ, Pfaller MA, Tenover FC, Yolken RH, editors. Manual Clin Microbiol. Washington, D.C: ASM Press; 1999. p. 442–50.

[11] CLSI. Performance Standards for Antimicrobial Susceptibility Testing. Clinical and Laboratory Standards Institute, Twenty-seventh Information Supplement, 2017; 37(1), M100-S26:1–249.

[12] Tarchouna M, Asma A, Ben-Selma W, Boukadida J (2013). Distribution of uropathogenic virulence genes in *Escherichia coli* isolated from patients with urinary tract infection. Inter J Infect Dis.

[13] Abiodun AO, Olufunke OA, Dunah FC, Oladiran F (2014). Phenotypic identification and phylogenetic characterization of Uropathogenic *Escherichia coli* in symptomatic pregnant women with urinary tract infections in South-Western Nigeria. Int J Biol.

[14] Mbanga J, Mudzana R (2014). Virulence factors and antibiotic resistance patterns of uropathogenic *Escherichia coli*. Afr J Microbiol Res.

[15] Derakhshandeh A, Firouzi R, Motamedifar M, Arabshahi S, Novinrooz A, Boroojeni AM (2015). Virulence characteristics and antibiotic resistance patterns among various phylogenetic groups of Uropathogenic *Escherichia coli* isolates. Jpn J Infect Dis.

[16] Usein CR, Damian M, Tatu-Chitoiu D, Capusa C, Fagaras R, Tudorache D (2001). Prevalence of virulence genes in *Escherichia coli* strains isolated from Romanian adult urinary tract infection cases. J Cell Mol Med.

[17] Le Bouguénec C, Archambaud M, Labigne A (1992). Rapid and specific detection of the *pap, afa*, and *sfa* adhesins - encoding operons in uropathogenic *Escherichia coli* strains by polymerase chain reaction. J Clin Microbiol.

[18] Johnson JR, Stell AL (2000). Extended virulence genotypes of *Escherichia coli* strains from patients with urosepsis in relation to phylogeny and host compromise. J Infect Dis.

[19] Le Bougue’nec C, Lalioui L, Du Merle L, Jouve M, courcoux P, Bouzari S, *et al.* Characterization of AfaE adhesins produced by extraintestinal and intestinal human *Escherichia coli* isolates. PCR assays for detection of Afa adhesions that do or do not recognize Dr blood group antigens. J Clin Microbiol 2001; 39:1738–1745. 10.1128/JCM.39.5.1738-1745.2001 PMC88018 11325983

[20] Bindereif A, Neilands JB (1985). Promoter mapping and transcriptional regulation of the iron assimilation system of plasmid ColV-K30 in *Escherichia coli* K-12. J Bacteriol.

[21] Molina-López J, Aparicio-Ozores G, Ribas-Aparicio RM, Gavilanes-Parra S, Chávez-Berrocal ME, Hernández-Castro R (2011). Drug resistance, serotypes, and phylogenetic groups among uropathogenic *Escherichia coli* including O25-ST131 in Mexico City. J Infect Dev Ctries.

[22] Clermont O, Bonacorsis BE (2000). Rapid and simple determination of *Escherichia coli* phylogenetic group. Appl Environ Microbiol.

[23] Clermont O, Christenson JK, Denamur E, Gordon DM (2013). The Clermont *Escherichia coli* phylo-typing method revisited: improvement of specificity and detection of new phylo-groups. Environ Microbiol Rep.

[24] Alm EW, Walk ST, Gordon DM, Walk ST, Feng PCH (2011). The niche of *Escherichia coli*. Population genetics of Bacteria.

[25] Themphachana M, Kongpheng S, Rattanachuay P, Khianngam S, Singkhamanan K, Sukhumungoon P (2016). Molecular characterization of virulence and antimicrobial susceptibility profiles of uropathogenic *Escherichia coli* from patients in a tertiary hospital, southern Thailand. Southeast Asian J Trop Med Public Health.

[26] Flores-Mireles AL, Walker JN, Caparon M, Hultgren SJ (2015). Urinary tract infections: epidemiology, mechanisms of infection and treatment options. Nat Rev Microbiol.

[27] Munkhdelger Y, Gunregjav N, Dorjpurev A, Juniichiro N, Sarantuya J (2017). Detection of virulence genes, phylogenetic group and antibiotic resistance of uropathogenic *Escherichia coli* in Mongolia. J Infect Dev Ctries.

[28] Momtaz H, Karimian A, Madani M, Safarpoor Dehkordi F, Ranjbar R, Sarshar M (2013). Uropathogenic *Escherichia coli* in Iran: serogroup distributions, virulence factors and antimicrobial resistance properties. Ann Clin Microbiol Antimicrob.

[29] Karimian A, Momtaz H, Mahbobe MM (2012). Detection of uropathogenic *Escherichia coli* virulence factors in patients with urinary tract infections in Iran. Afr J Microbiol Res.

[30] Wang Y, Zhao S, Han L, Guo X, Chen M, Ni Y (2014). Drug resistance and virulence of uropathogenic Escherichia coli from Shanghai, China. J Antibiot.

[31] Paniagua-Contreras Gloria Luz, Monroy-Pérez Eric, Rodríguez-Moctezuma José Raymundo, Domínguez-Trejo Pablo, Vaca-Paniagua Felipe, Vaca Sergio (2017). Virulence factors, antibiotic resistance phenotypes and O-serogroups of Escherichia coli strains isolated from community-acquired urinary tract infection patients in Mexico. Journal of Microbiology, Immunology and Infection.

[32] Santo E, Macedo C, Marin JM (2006). Virulence factors of uropathogenic *Escherichia coli* from a University Hospital in Ribeirão Preto, São Paulo, Brazil. Rev Inst Med trop S Paulo.

[33] Rahdar M, Rashki A, Miri HR, Ghalehnoo MR (2015). Detection of *pap*, *sfa*, *afa*, *foc,* and *fim* Adhesin-encoding operons in Uropathogenic *Escherichia coli* isolates collected from patients with urinary tract infection. Jundishapur J Microbiol.

[34] Alabsi MS, Ghazal A, Sabry SA, Alasaly MM (2014). Association of some virulence genes with antibiotic resistance among uropathogenic *Escherichia coli* isolated from urinary tract infection patients in Alexandria, Egypt: A hospital-based study. J Glob Antimicrob Resist.

[35] Bashir S, Haque A, Sawar Y, Ali A, Anwar MI (2012). Virulence profile of different phylogenetic groups of locally isolated community acquired uropathogenic *E. coli* from Faisalabad region of Pakistan. Ann Clin Microbiol Antimicrob.

[36] Jalali HR, Eslami G, Fallah F, Pourbakhsh A (2015). Genotyping of virulence factors of Uropathogenic *Escherichia coli* by PCR. NovelBiomed..

[37] Merza NS, Jubrael JMS (2015). The prevalence of virulence factors among Uropathogenic *Escherichia coli* strains isolated from different hospitals in Kurdistan region-Iraq. Int J Bioinform Biomed Eng.

[38] Ejrnæs K, Stegger M, Reisner A, Ferry S, Monsen T, Stig EH (2011). Characteristics of *Escherichia coli* causing persistence or relapse of urinary tract infections: phylogenetic groups, virulence factors and biofilm formation. Virulence.

[39] Lee JH, Subhadra B, Son YJ, Kim DH, Park HS, Kim JM (2016). Phylogenetic group distributions, virulence factors and antimicrobial resistance properties of uropathogenic *Escherichia coli* strains isolated from patients with urinary tract infections in South Korea. Lett Appl Microbiol.

[40] Bien Justyna, Sokolova Olga, Bozko Przemyslaw (2012). Role of UropathogenicEscherichia coliVirulence Factors in Development of Urinary Tract Infection and Kidney Damage. International Journal of Nephrology.

[41] Garcia TA, Ventura CL, Smith MA, Merrell DS, O’Brien AD (2013). Cytotoxic necrotizing factor 1 and hemolysin from uropathogenic *Escherichia coli* elicit different host responses in the murine bladder. Infect Immun.

[42] Kot B, Wicha J, Gruzewska A, Piechota M, Wolska K, Obrebska M (2016). Virulence factors, biofilm-forming ability, and antimicrobial resistance of urinary *Escherichia coli* strains isolated from hospitalized patients. Turk J Med Sci.

[43] Yun KW, Kim HY, Park HK, Kim W, Lim IS (2014). Virulence factors of uropathogenic *Escherichia coli* of urinary tract infections and asymptomatic bacteriuria in children. J Microbiol Immun Infect.

[44] Sivick KE, Mobley HL (2010). Waging war against uropathogenic *Escherichia coli*: winning back the urinary tract. Infect Immun.

[45] Tabasi M, Karam MRA, Habibi M, Mostafavi E, Bouzari S (2016). Genotypic characterization of virulence factors in *Escherichia coli* isolated from patients with acute cystitis, pyelonephritis and asymptomatic Bacteriuria. J Clin Diagn Res.

[46] Garenaux A, Caza M, Dozoise CM (2011). The ins and out of siderophore mediated uptake by extra-intestinal pathogenic Escherichia coli. Vet Microbiol.

[47] Iranpour Darioush, Hassanpour Mojtaba, Ansari Hossein, Tajbakhsh Saeed, Khamisipour Gholamreza, Najafi Akram (2015). Phylogenetic Groups ofEscherichia coliStrains from Patients with Urinary Tract Infection in Iran Based on the New Clermont Phylotyping Method. BioMed Research International.

[48] Grude N, Potaturkina-Nesterova NI, Jenkins A, Strand L, Nowrouzian FL, Nyhus J (2007). A comparison of phylogenetic group, virulence factors and antibiotic resistance in Russian and Norwegian isolates of *Escherichia coli* from urinary tract infection. Clin Microbiol Infect.

[49] Gao Q, Zhang D, Ye Z, Zhu X, Yang W, Dong L (2017). Virulence traits and pathogenicity of uropathogenic *Escherichia coli* isolates with common and uncommon O serotypes. Microb Pathog.

[50] Derakhshandeh A, Firouzi R, Moatamedifar M, Motamedi A, Bahadori M, Naziri Z (2013). Phylogenetic analysis of *Escherichia coli* strains isolated from human samples. Mol Biol Res Commun.

[51] Johnson JR, Kuskowski MA, Gajewski A, Soto S, Horcajada JP, Jimenez de Anta MT (2005). Extended virulence genotypes and phylogenetic background of *Escherichia coli* isolates from patients with cystitis, pyelonephritis or prostatitis. J Infect Dis.

[52] Regasa Dadi B, Abebe T, Zhang L, Mihret A, Abebe W, Amogne W (2018). Drug resistance and plasmid profile of uropathogenic *Escherichia coli* among urinary tract infection patients in Addis Abeba. J Infect Dev Ctries.

[53] Anusha SU, Sundar SK, Rajan S (2015). RAPD pattern, virulence nature and plasmid profile of MDR uropathogenic *Escherichia coli*. Adv Appl Sci Res.

[54] Akingbade O, Balogun S, Ojo D, Akinduti P, Okerentugba PO, Nwanze JC (2014). Resistant plasmid profile analysis of multidrug resistant *Escherichia coli* isolated from urinary tract infections in Abeokuta, Nigeria. Afr Health Sci.

[55] Annapurna YV, Reddy BS, Lakshmi VV (2014). Multidrug resistance and virulence phenotypes among Uropathogenic *Escherichia coli*. Int J Curr Microbiol App Sci.

[56] Gizachew M, Kebede M, Merid Y, Sinshaw Y, Tiruneh M, Alemayehu M (2013). Escherichia coli isolated from patients suspected for urinary tract infections in Hawassa referral hospital, southern Ethiopia: an institution based cross sectional study. E3 J Microbiol Res.

[57] FMHACA. Standard Treatment Guidelines For General Hospital. Food, Medicine and Healthcare Administration and Control Authority of Ethiopia, 3rd Edn, pp. 2014; 233–7.

[58] Abdu A, Kachallah M, Bolus DY (2018). Antibiotic susceptibility patterns of Uropathogenic Escherichia coli among patients with urinary tract infections in a tertiary care hospital in Maiduguri, north eastern, Nigeria. J Biosci Biotechnol Discov.

